# Robust IgM responses following intravenous vaccination with Bacille Calmette–Guérin associate with prevention of *Mycobacterium tuberculosis* infection in macaques

**DOI:** 10.1038/s41590-021-01066-1

**Published:** 2021-11-22

**Authors:** Edward B. Irvine, Anthony O’Neil, Patricia A. Darrah, Sally Shin, Alok Choudhary, Wenjun Li, William Honnen, Smriti Mehra, Deepak Kaushal, Hannah Priyadarshini Gideon, JoAnne L. Flynn, Mario Roederer, Robert A. Seder, Abraham Pinter, Sarah Fortune, Galit Alter

**Affiliations:** 1 Ragon Institute of MGH, MIT and Harvard, Cambridge, MA USA; 2 Department of Immunology and Infectious Diseases, Harvard T.H. Chan School of Public Health, Boston, MA USA; 3 Vaccine Research Center, National Institute of Allergy and Infectious Diseases (NIAID), National Institutes of Health, Bethesda, MD USA; 4 Public Health Research Institute, New Jersey Medical School, Rutgers, The State University of New Jersey, Newark, NJ USA; 5 Department of Medicine, University of Massachusetts Medical School, Worcester, MA USA; 6 Division of Microbiology, Tulane National Primate Research Center, Covington, LA USA; 7 Southwest National Primate Research Center, Texas Biomedical Research Institute, San Antonio, TX USA; 8 Department of Microbiology and Molecular Genetics and Center for Vaccine Research, University of Pittsburgh School of Medicine, Pittsburgh, PA USA

**Keywords:** Vaccines, Tuberculosis, Antibodies

## Abstract

Development of an effective tuberculosis (TB) vaccine has suffered from an incomplete understanding of the correlates of protection against *Mycobacterium tuberculosis* (*Mtb*). Intravenous (i.v.) vaccination with Bacille Calmette–Guérin (BCG) provides nearly complete protection against TB in rhesus macaques, but the antibody response it elicits remains incompletely defined. Here we show that i.v. BCG drives superior antibody responses in the plasma and the lungs of rhesus macaques compared to traditional intradermal BCG administration. While i.v. BCG broadly expands antibody titers and functions, IgM titers in the plasma and lungs of immunized macaques are among the strongest markers of reduced bacterial burden. IgM was also enriched in macaques that received protective vaccination with an attenuated strain of *Mtb*. Finally, an *Mtb*-specific IgM monoclonal antibody reduced *Mtb* survival in vitro. Collectively, these data highlight the potential importance of IgM responses as a marker and mediator of protection against TB.

## Abstract (web summary)

Alter and colleagues show that IgM titers in the plasma and bronchoalveolar lavage fluid represent markers of reduced Mtb burden in rhesus macaques vaccinated intravenously with Bacille Calmette–Guérin.

## Main

*Mtb*, the causative agent of TB, was responsible for the death of an estimated 1.4 million individuals in 2019 (ref. ^1^). While TB is curable, the intensive antibiotic regimen coupled with the rise in antibiotic resistance underscores the need for an efficacious vaccine to help mitigate the global TB epidemic. BCG, the current standard for TB vaccination, is effective at preventing severe forms of TB in young children, but does not reliably prevent pulmonary TB in adults^2^.

TB vaccine development has suffered from a lack of understanding of the determinants of immunity against *Mtb* infection. Studies in CD4^+^ T cell-deficient mice^3^ and nonhuman primates^4,5^, as well as studies in humans infected with human immunodeficiency virus (HIV)^6^, have found increased rates of TB disease progression in the setting of low CD4^+^ T cell counts, indicating a critical role for CD4^+^ T cells in controlling *Mtb* infection. However, efforts to generate vaccines that primarily leverage T cell immunity to drive protection against TB have been met with limited success. Although the phase 2b type 1 helper T (T_H_1) cell-directed MVA-85A vaccine trial failed^7^, a post hoc correlation analysis suggested that Ag85A-specific IgG responses were linked with reduced risk of TB disease^8^. These data pointed to humoral immunity as a potential negative correlate of TB disease risk. More recently, the phase 2b trial of the M72/AS01_E_ TB vaccine in adults reported a 49.7% reduction in the rate of progression to active TB in vaccinees compared to the placebo group^9^. While robust T cell immunity was present following M72 vaccination, strong M72-specific humoral immunity was also observed^9^.

In addition to the efficacy signals emerging in human TB vaccine studies^9,10^, i.v. BCG vaccination provides robust protection against *Mtb* infection in rhesus macaques^11^. Specifically, i.v. BCG immunization resulted in a 100,000-fold reduction in lung bacterial burden compared with standard-dose intradermal (i.d.) BCG vaccination, with six of ten macaques showing no detectable *Mtb* infection^11^. Intravenous BCG vaccination led to a marked increase in antigen-responsive T cells in the blood and bronchoalveolar lavage (BAL) fluid compared to standard-dose i.d. BCG vaccination. Concomitant with enhanced T cell responses, i.v. BCG immunization elicited robust whole-cell lysate-reactive antibody responses in the plasma and BAL^11^. However, the antigenic targets, functional and antimicrobial activity and relationship with *Mtb* burden of the humoral responses triggered by i.v. BCG vaccination were not defined.

Here we investigated the antigen-specific humoral immune responses induced by i.v. BCG vaccination in rhesus macaques. We show that compared to standard-dose i.d. BCG, i.v. BCG vaccination elicited superior antigen-specific humoral immunity in the periphery, and uniquely induced a robust, lung-compartmentalized antibody response able to restrict *Mtb* growth in vitro. While IgG, IgA and several Fc receptor-binding antibody subpopulations expanded selectively in the lungs of i.v.-immunized macaques, antigen-specific IgM titers in the plasma and BAL strongly associated with reduced bacterial burden. Finally, an *Mtb*-specific IgM monoclonal antibody reduced *Mtb* survival in a whole-blood model of infection, suggesting that vaccine-induced IgM responses may contribute to vaccine-induced protection against *Mtb*.

## Results

### Intravenous BCG drives high and durable plasma antibody titers

**Fig. 1:**
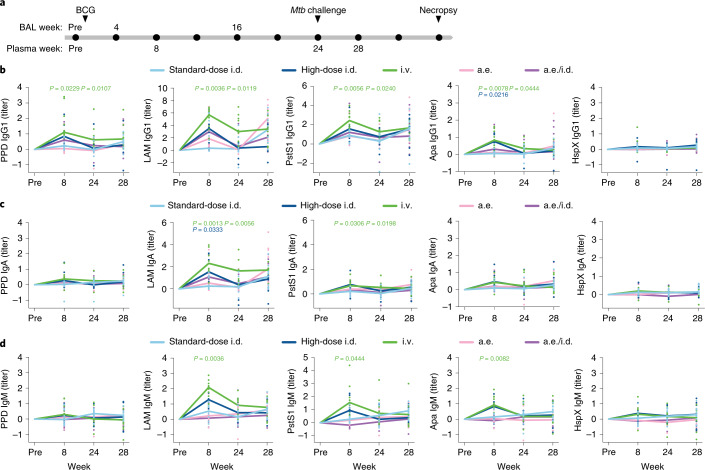
Intravenous BCG-immunized macaques exhibit higher and more durable plasma antibody titers. **a**, Timeline of the original vaccination study indicating the samples available by compartment. Week 12 rather than week 16 BAL samples were analyzed in four of eight macaques in the high-dose i.d. group. BAL samples from all remaining macaques were analyzed at week 16. **b**–**d**, Fold change in IgG1 (**b**), IgA (**c**) and IgM (**d**) titers present in the plasma of each rhesus macaque following BCG vaccination. Fold changes were calculated as fold change in Luminex median fluorescence intensity (MFI) over the pre-vaccination level for each macaque. A base-2 log scale was used for the *y* axis. Each point represents the duplicate average from a single macaque. The lines show group medians over time. Kruskal–Wallis with Dunn’s multiple-comparison tests were performed on the fold change values at each time point, comparing each experimental BCG vaccination group to the standard-dose i.d. BCG group. Adjusted *P* values < 0.05 compared to the standard-dose i.d. BCG group are shown and are colored by vaccination group. Source data

Rhesus macaques were immunized with standard-dose i.d. BCG (*n* = 10), high-dose i.d. BCG (*n* = 8), i.v. BCG (*n* = 10), aerosol (a.e.) BCG (*n* = 10) or a combination of a.e. and i.d. (a.e./i.d.) BCG (*n* = 10)^11^. Plasma was collected before vaccination, as well as weeks 8, 24 and 28 after BCG vaccination (Fig. 1a). BAL was collected before vaccination, as well as weeks 4 and 16 after BCG vaccination (Fig. 1a). The immunized macaques were challenged with *Mtb* at week 24 after BCG vaccination (Fig. 1a). Due to concerns that the BAL sampling could perturb the course of infection after challenge, only plasma was collected following *Mtb* challenge at week 28. To determine whether particular antigen-specific antibody populations were differentially induced by different BCG vaccination strategies, we used a custom, multiplexed Luminex assay^12^. Antibody titers were assessed to five *Mtb* antigens that vary in composition, structure, function and localization: purified protein derivative (PPD), the total protein fraction from virulent mycobacterium^13^, lipoarabinomannan (LAM), a critical cell wall glycolipid^14^, HspX, a stress-induced intracellular protein^15^, and PstS1 and Apa, cell membrane-associated glycoproteins linked to host cell invasion^16,17^. Of note, antibodies specific to LAM, HspX and PstS1 ameliorate TB disease in mouse passive transfer studies^18–20^, and antibody responses to each of the five antigens have been observed during *Mtb* infection in humans^20–22^.

Titers for PPD, LAM, PstS1 and Apa IgG1 antibodies in the i.v. BCG group were each significantly higher than those in the standard-dose i.d. BCG group both at week 8 and week 24 (*Mtb* challenge) (Fig. 1b). Macaques in the high-dose i.d. and a.e./i.d. BCG groups trended toward higher IgG1 levels to these four antigens compared to the standard-dose i.d. BCG group, but this difference was only significant for the Apa-specific response in the high-dose i.d. BCG group (Fig. 1b). Macaques that received BCG vaccination by the i.v. route maintained stable titers of LAM IgG1 in the plasma between week 24 and week 28 (4 weeks after *Mtb*), whereas macaques that received BCG vaccination by the i.d. (standard dose), a.e. and a.e./i.d. routes all displayed an increase in LAM IgG1 titers compared to their respective levels at week 24 (Fig. 1b), perhaps reflective of poor control of *Mtb* replication in these groups compared to the i.v. BCG vaccination group^11^. Macaques vaccinated i.v. with BCG exhibited significantly higher plasma IgA titers to LAM and PstS1 at week 8 and week 24 compared to macaques vaccinated with standard-dose BCG i.d. (Fig. 1c). Macaques vaccinated i.d with high-dose BCG also had a significant increase in LAM IgA titers at week 8 after vaccination compared to macaques vaccinated with standard-dose BCG i.d. (Fig. 1c).

Consistent with IgG1 and IgA responses, the i.v. BCG group mounted the strongest plasma IgM response to vaccination, with significantly higher LAM-specific, PstS1-specific and Apa-specific IgM titers at week 8 after vaccination, and higher (although not statistically significant) LAM IgM titers at week 24 compared to the standard-dose i.d. BCG group (Fig. 1d). High-dose i.d. BCG-vaccinated macaques also exhibited a trend toward increased IgM titers to LAM, PstS1 and Apa over standard-dose i.d. BCG-vaccinated macaques; however, these differences were not statistically significant (Fig. 1d). These data indicate that, in contrast to standard-dose i.d. BCG immunization of rhesus macaques, i.v. BCG immunization resulted in robust antigen-specific antibody titers in the plasma, with protein-specific and LAM-specific antibody responses persisting in the i.v. group up to the time of *Mtb* challenge.

### Intravenous BCG elicits a robust antibody response in the lungs

**Fig. 2:**
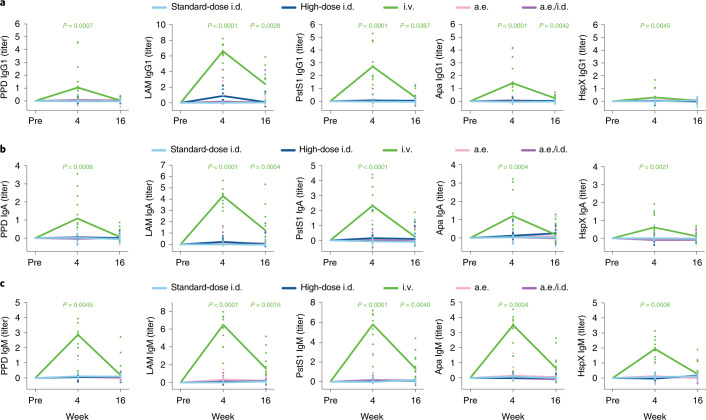
Intravenous BCG vaccination uniquely elicits a robust lung-compartmentalized antibody response. **a**–**c**, Fold change values in IgG1 (**a**), IgA (**b**) and IgM (**c**) titers present in the BAL fluid of each rhesus macaque following BCG vaccination. Fold changes were calculated as the fold change in Luminex MFI over the pre-vaccination level for each macaque. A base-2 log scale was used for the *y* axis. Each point represents the duplicate average from a single macaque. The lines represent group medians over time. Kruskal–Wallis with Dunn’s multiple-comparison tests were performed on the fold change values at each time point, comparing each experimental BCG vaccination group to the standard-dose i.d. BCG group. Adjusted *P* values < 0.05 compared to the standard-dose i.d. BCG group are shown and are colored by vaccination group. Source data

We next profiled the antibody response at the site of infection. BAL fluid was collected before vaccination, as well as at weeks 4 and 16 after BCG vaccination (Fig. 1a). At week 4, i.v. BCG-immunized macaques showed robust IgG1, IgA and IgM responses in the BAL samples that were significantly higher than those triggered by standard-dose i.d. BCG vaccination across all antigens tested (Fig. 2a–c), with a 100-fold increase in antibody titers over pre-vaccination levels observed in some macaques vaccinated by the i.v. route (Fig. 2a). Some of these responses were transient, but significantly higher levels of LAM IgG1, PstS1 IgG1, Apa IgG1, LAM IgA, LAM IgM and PstS1 IgM were observed in the i.v. BCG group versus the standard-dose i.d. BCG group at week 16 (Fig. 2a–c). These data demonstrate that i.v. BCG vaccination alone induced a strong, lung-compartmentalized BCG-specific antibody response, which persisted at detectable levels for at least 4 months following vaccination.

### Intravenous BCG-induced antibodies mediate innate immune activation

**Fig. 3:**
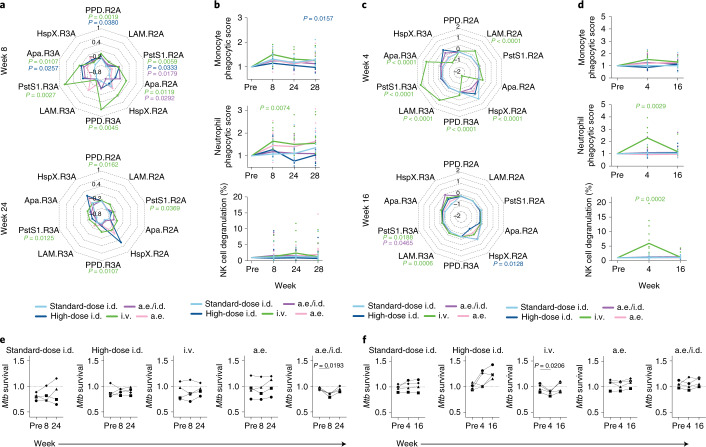
Antibodies from intravenous BCG-vaccinated macaques drive innate immune activation. **a**,**c**, Radar plots of fold changes in plasma (**a**) and BAL (**c**) antibody FcγR binding activity of each group after BCG vaccination. Fold changes were calculated as the fold change in Luminex MFI over the pre-vaccination level for each macaque. Median *z*-scores of each group are plotted. **b**,**d**, Fold change values in plasma (**b**) and BAL (**d**) antibody-dependent cellular phagocytosis (ADCP) by THP-1 cells (top), ADNP by primary human neutrophils (middle) and antibody-dependent primary human NK cell degranulation determined by the percentage of CD107a-positive cells (bottom). Fold changes were calculated as the fold change over the pre-vaccination level for each macaque. Each point represents the duplicate average from a single macaque. The lines represent group medians over time. **e**,**f**, Macrophage restriction assay using pooled plasma (**e**) and BAL (**f**) from each vaccination group at each time point. The *y* axis (*Mtb* survival) shows live (GFP)/total (mCherry) *Mtb* burden in human MDMs normalized by the no-antibody condition for the respective donor. Each point is the triplicate average from one healthy human macrophage donor. Each donor is represented by a unique marker shape. **a**–**d**, Kruskal–Wallis with Dunn’s multiple-comparison test was performed on the fold change values at each time point, comparing each experimental BCG vaccination group to the standard-dose i.d. BCG group. Adjusted *P* values < 0.05 compared to the standard-dose i.d. BCG group are shown and are colored by vaccination group. **e**,**f**, Repeated-measures one-way analysis of variance (ANOVA) with Dunnett’s multiple-comparisons test was performed for each vaccination group, comparing pre-vaccination restrictive activity with that of each post-vaccination time point. Adjusted *P* values < 0.05 are shown. Source data

**Extended Data Fig. 1:**
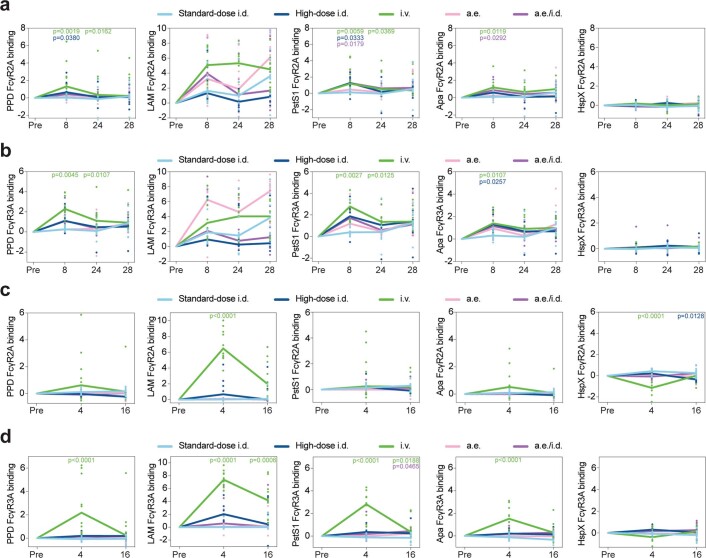
Antigen-specific FcγR binding capacity of plasma and BAL antibodies. Fold change in **a** and **c**, FcγR2A, and **b** and **d**, FcγR3A binding antibodies to PPD, LAM, PstS1, Apa, and HspX in the **a** and **b**, plasma, and **c** and **d**, BAL of each BCG-vaccinated rhesus macaque. Fold changes were calculated as fold change in Luminex median fluorescence intensity (MFI) over the pre-vaccination level for each macaque. A base-2 log scale is used for the y-axis. Each point represents the duplicate average from a single macaque. The lines show group medians over time. Kruskal-Wallis with Dunn’s multiple-comparison tests were performed on the fold change values at each timepoint, comparing each experimental BCG vaccination group to the standard-dose i.d. BCG group. Adjusted p-values < 0.05 compared to the standard-dose i.d. BCG group are shown and are colored by vaccination group. Source data

Beyond their ability to bind and recognize pathogens or pathogen-infected cells, antibodies deploy the antimicrobial activity of the innate immune system through Fc receptor engagement^23^. Thus, we measured the Fc gamma receptor (FcγR) binding and functional capacity of plasma-derived and BAL-derived antibodies elicited by each BCG vaccination strategy. The i.v. BCG group showed significantly higher levels of FcγR2A-binding and FcγR3A-binding antibodies for PPD, PstS1 and Apa in the plasma at week 8 compared to the standard-dose i.d. BCG group (Fig. 3a and Extended Data Fig. 1). Further, macaques vaccinated i.v. with BCG maintained significantly higher PPD-specific and PstS1-specific FcγR2A-binding and FcγR3A-binding antibodies in the plasma at week 24 (*Mtb* challenge) compared to standard-dose i.d. BCG-vaccinated macaques (Fig. 3a and Extended Data Fig. 1). To examine the Fc functionality of LAM-specific antibodies in the plasma, we performed antibody-dependent phagocytosis assays with monocytes and neutrophils, and degranulation assays using natural killer (NK) cells, a surrogate for antibody-dependent cellular cytotoxicity^24^. Plasma LAM-specific antibodies from macaques vaccinated i.v. with BCG induced the most potent antibody-dependent neutrophil phagocytosis (ADNP), which was (moderately) significantly higher than that observed for the LAM-specific antibodies in the standard-dose i.d. group at week 8 (Fig. 3b). Limited differences in plasma LAM-specific antibody-dependent monocyte phagocytosis and antibody-dependent NK cell degranulation were present between vaccine groups (Fig. 3b).

Intravenous BCG-vaccinated macaques had significantly higher amounts of LAM-specific FcγR2A-binding antibodies in the BAL fluid at week 4 compared to standard-dose i.d. BCG-vaccinated macaques (Fig. 3c and Extended Data Fig. 1). In addition, antigen-specific FcγR3A binding in the BAL fluid was particularly robust in the i.v. BCG group, with significantly higher amounts of FcγR3A-binding antibodies to PPD, LAM, PstS1 and Apa at week 4 as compared to the standard-dose i.d. BCG group (Fig. 3c and Extended Data Fig. 1). LAM-specific and PstS1-specific FcγR3A-binding antibody titers remained significantly higher in the i.v. BCG group compared to the standard-dose i.d. BCG group at week 16 (Fig. 3c and Extended Data Fig. 1). Furthermore, LAM-specific antibodies in the BAL of i.v. BCG-vaccinated macaques showed a trend toward stronger antibody-dependent monocyte phagocytosis activity, and significantly higher ADNP and NK cell degranulation activity at week 4 as compared to LAM-specific antibodies in the BAL of standard-dose i.d. BCG-vaccinated macaques (Fig. 3d). This LAM-specific antibody functionality in the i.v. BCG group returned to baseline levels by week 16 (Fig. 3d). BAL-derived LAM-specific antibodies in the standard-dose i.d., high-dose i.d., a.e. and a.e./i.d. BCG groups exhibited little functionality at weeks 4 and 16 (Fig. 3d).

Because an enrichment in FcγR3A-binding and NK cell-activating antibodies in the setting of latent TB has been linked to enhanced intracellular *Mtb* killing in macrophages^25^, we examined the antimicrobial activity of antibodies from all vaccination groups. Human monocyte-derived macrophages (MDMs) from four distinct donors were infected with an H37Rv live/dead fluorescent reporter strain of *Mtb* (*Mtb-*live/dead, GFP/mCherry)^26^. Following infection, macrophages were incubated with pooled plasma or BAL samples from each vaccination group. Plasma from the i.v. BCG group at weeks 8 and 24 did not drive significant *Mtb* restriction as compared to i.v. plasma from the pre-vaccination time point (Fig. 3e). However, BAL samples at week 4 from the i.v. BCG group drove moderate, yet significant, *Mtb* restriction in macrophages as compared to i.v. BAL from the pre-vaccination time point (Fig. 3f). Of the remaining vaccination groups, only plasma at week 4 from the a.e./i.d. BCG group mediated significant *Mtb* restriction in macrophages as compared to the pre-vaccination time point plasma (Fig. 3e). These observations indicate that i.v. BCG vaccination elicits a functional antibody response, with a selective increase in FcγR3A binding, NK cell degranulation and intracellular *Mtb* killing in macrophages mediated by antibodies in the BAL fluid of these macaques.

### IgM titers negatively correlate with *Mtb* burden

**Fig. 4:**
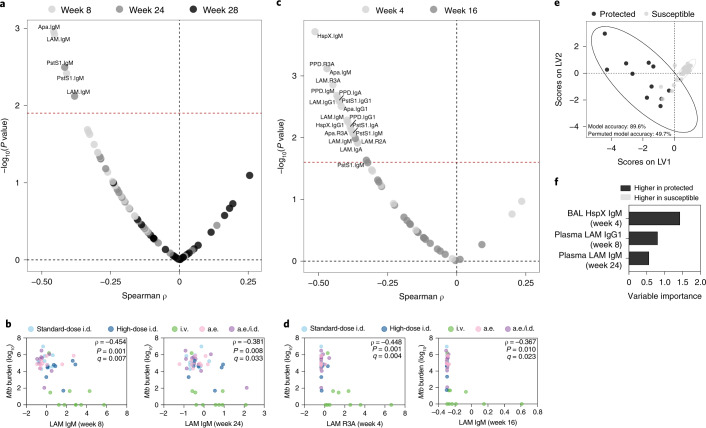
Numerous BCG-induced antibody features are associated with reduced *Mtb* burden. **a**,**c**, Spearman correlations between base-10 log(*Mtb* burden) at necropsy and each plasma (**a**) and BAL (**c**) antibody measurement after vaccination. Labeled features above the red dashed line have an adjusted *P* value (*q* value) < 0.05 by the Benjamini–Hochberg procedure. **b**,**d**, Spearman correlations between base-10 log(*Mtb* burden) at necropsy and select plasma (**b**) and BAL (**d**) antibody measurements. Fold change antibody measurements were subjected to a *z*-score transformation before plotting. The *q* values were computed by the Benjamini–Hochberg procedure. **e**,**f**, PLS-DA model fit using the antibody features selected by LASSO regularization. **e**, Graph of the first two latent variables (LVs) of the model. Protected macaques (black) had an *Mtb* burden < 1,000 colony-forming units at necropsy. Susceptible macaques (gray) had an *Mtb* burden > 1,000 colony-forming units at necropsy. Ellipses show the 95% confidence intervals. Two-tailed Mann–Whitney *U* test, *P* < 2.2 × 10^−16^. **f**, Variable importance in the projection (VIP) coefficients on LV1 for each feature. Source data

Next, we combined the vaccination groups and queried whether specific features of the antibody response correlated with *Mtb* burden in the lung measured at necropsy^11^. In the plasma, LAM-, PstS1- and Apa-specific IgM titers at week 8, as well as LAM- and PstS1-specific IgM titers at week 24 significantly negatively correlated with *Mtb* burden in the lung (Fig. 4a,b). We did not detect plasma titers or features that significantly positively associated with *Mtb* burden (Fig. 4a). In the BAL, 18 antibody features significantly negatively correlated with *Mtb* burden in the lung (Fig. 4c). These included BAL IgG1, IgA and IgM specific to LAM and PstS1, as well as BAL LAM-, PstS1- and Apa-specific FcγR-binding antibodies (Fig. 4c,d). These features were all week 4 measurements with the exception of week 16 LAM-specific and PstS1-specific IgM titers (Fig. 4c,d). No BAL antibody features had a significant positive correlation with *Mtb* burden (Fig. 4c). Collectively, these data indicate that several humoral features negatively correlate with lung *Mtb* burden in this cohort; however, IgM responses alone tracked with reduced *Mtb* burden close to the time of challenge across both compartments (plasma at week 24 and BAL at week 16).

### Antibody profiles distinguish vaccine-induced protection

We next sought to determine whether a minimal set of antibody features that collectively tracked with *Mtb* controls could be defined. Macaques with a lung *Mtb* burden at necropsy below 1,000 colony-forming units (nine i.v. BCG, one high-dose i.d. BCG, one a.e./i.d. BCG; total *n* = 11) were categorized as protected, while those with an *Mtb* burden greater than or equal to 1,000 colony-forming units were categorized as susceptible (total *n* = 37). Next, least absolute shrinkage and selection operator (LASSO) regularization was implemented on the standardized antibody data, removing variables unrelated to the outcome, as well as reducing the number of highly correlated features. We then performed partial least-squares discriminant analysis (PLS-DA) to visualize and quantify group separation. A robust separation between protected and susceptible macaques was observed on the basis of antibody profile (Fig. 4e). The model distinguished protected from susceptible macaques with a balanced cross-validation accuracy of 89.6% (Fig. 4e). Only three features were required to achieve this high level of predictive accuracy: BAL HspX-specific IgM at week 4, plasma LAM-specific IgG1 at week 8 and plasma LAM-specific IgM at week 24 (Fig. 4f). The selection of these three variables across distinct time points suggests that substantive antibody differences were present between protected and susceptible macaques from week 4 to week 24 after vaccination that can be used to accurately resolve vaccine-induced protection.

### IgM titers are enriched following *Mtb-ΔsigH* vaccination

**Fig. 5:**
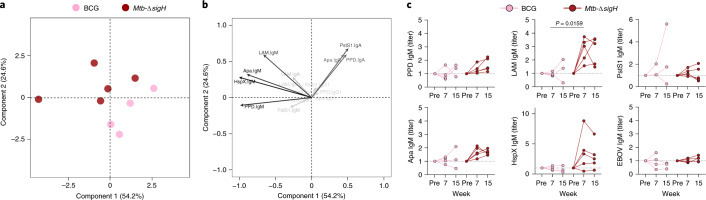
Protective vaccination with attenuated *Mtb* (*Mtb-ΔsigH*) is associated with increased plasma IgM titers. **a**,**b**, PCA using fold change IgG1, IgA and IgM titers measured at week 7 after vaccination in an attenuated *Mtb* rhesus macaque vaccination cohort. Fold changes were calculated as the fold change in Luminex MFI over the pre-vaccination level for each macaque. **a**, PCA score plot. **b**, PCA loading plot. Relative contribution of variables to the components are indicated by a color gradient. Light-gray variables contribute least, and black variables contribute most. **c**, Fold change in IgM titers present in the plasma of each rhesus macaque following vaccination. Each point represents the duplicate average from a single macaque. *Mtb* challenge was performed at week 8 after vaccination. Two-tailed Mann–Whitney *U* tests were performed on the fold change values at each time point, comparing the *Mtb-ΔsigH* to the BCG group. *P* values < 0.05 are shown. Source data

**Extended Data Fig. 2:**
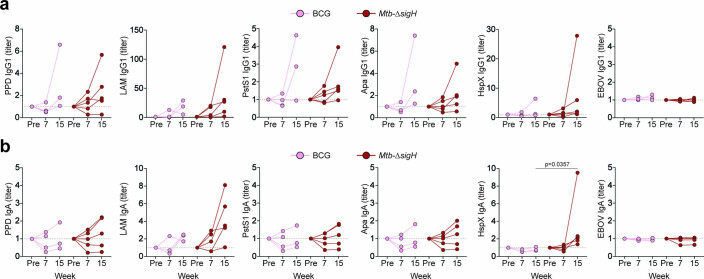
Plasma IgG1 and IgA titers from the attenuated *Mtb* (*Mtb-ΔsigH*) vaccination cohort. Fold change in **a**, IgG1 and **b**, IgA titers present in the plasma of each rhesus macaque following vaccination. Fold changes were calculated as fold change in Luminex MFI over the pre-vaccination level for each macaque. Each point represents the duplicate average from a single macaque. *Mtb* challenge was performed week 8 post-vaccination. Two-tailed Mann-Whitney U tests were performed on the fold change values at each timepoint, comparing the *Mtb-ΔsigH* to the BCG group. P-values < 0.05 are shown. Source data

Because vaccination route was closely linked to protection in the BCG vaccination cohort^11^, the generalizability of the associations with reduced *Mtb* burden was unclear. Thus, we next investigated whether similar antibody features were associated with control of *Mtb* burden in an independent vaccination study. Because a.e. vaccination with the attenuated *Mtb* strain, *Mtb-ΔsigH*, was reported to provide superior protection compared to a.e. BCG vaccination in rhesus macaques^27^, we performed antibody profiling on the plasma of macaques vaccinated a.e. with *Mtb-ΔsigH* (*n* = 5) or a.e. with BCG (*n* = 4). Focusing on peak antibody titer measurements, the *Mtb-ΔsigH* and BCG groups could be separated by principal-component analysis (PCA; Fig. 5a). Analysis of the loadings plot indicated that IgM responses were enriched in *Mtb-ΔsigH*-vaccinated macaques as compared to a.e. BCG-vaccinated macaques (Fig. 5b). Similarly, univariate analyses indicated that a.e. *Mtb-ΔsigH*-vaccinated macaques elicited significantly higher LAM-specific IgM titers, as well as a trend toward increased Apa-specific and HspX-specific IgM titers as compared to a.e. BCG-vaccinated macaques (Fig. 5c). By contrast, minimal differences in antigen-specific IgG1 and IgA titers were observed between the *Mtb-ΔsigH* and BCG vaccination groups (Extended Data Fig. 2). These data demonstrate that increased plasma IgM titers also tracked with reduced *Mtb* burden in an orthogonal rhesus macaque vaccination study, potentially indicating a common association between antigen-specific IgM and vaccine-induced *Mtb* control.

### LAM-specific IgM drives increased *Mtb* restriction in vitro

**Fig. 6:**
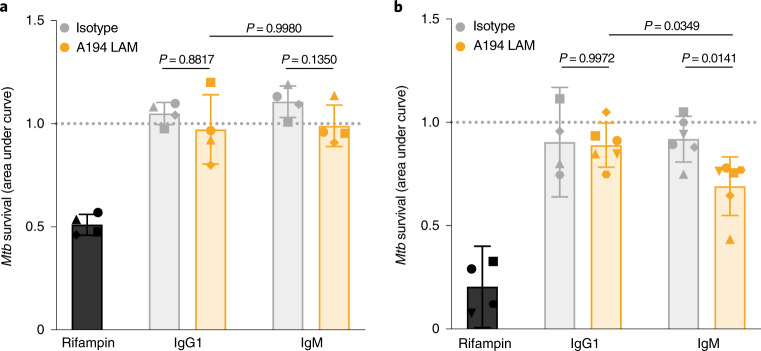
LAM IgM monoclonal antibody drives superior *Mtb* restriction in human whole blood. **a**, Macrophage restriction assay. Each treatment (50 µg ml^−1^ antibody; 1 µg ml^−1^ rifampin) was added to human MDMs infected with *Mtb-276*, a luciferase reporter strain for *Mtb* in the H37Rv genetic background. Growth curves in the presence of each antibody treatment were generated by taking luminescence readings every 24 h up to 96 h. The *y* axis (*Mtb* survival) is the area under the *Mtb* growth curve normalized by the no-antibody condition of each donor. Each point is the triplicate average from one donor. Each donor is represented by a unique marker shape. Error bars denote the mean with standard deviation. Repeated-measures one-way ANOVA with Sidak’s multiple-comparisons test was performed. *P* values of relevant comparisons are shown. **b**, Whole-blood restriction assay. Each antibody (25 µg ml^−1^) was tested for its ability to drive *Mtb* restriction in the context of fresh human whole blood using *Mtb-276*. The *y* axis (*Mtb* survival) is the area under the *Mtb* growth curve, normalized by the no-antibody condition of each donor. Growth curves were generated by taking luminescence readings every 24 h up to 120 h. Each point is the triplicate average from one donor. Error bars denote the mean with standard deviation. Ordinary one-way ANOVA with Sidak’s multiple-comparisons test was performed. *P* values of relevant comparisons are shown. Source data

**Extended Data Fig. 3:**
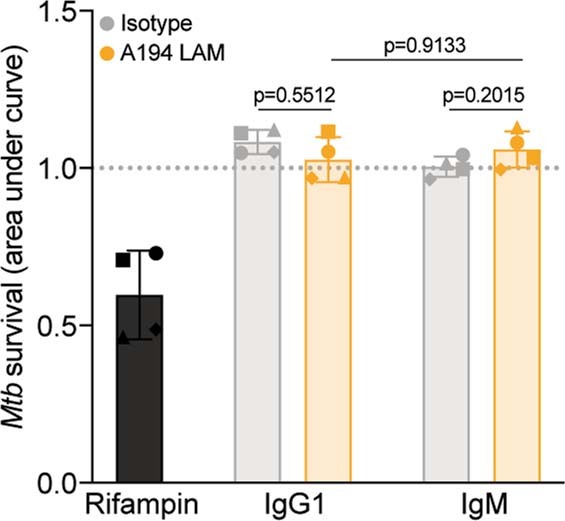
LAM IgM monoclonal antibody does not drive significant *Mtb* restriction in macrophages when added prior to infection. Macrophage restriction assay. *Mtb-276* was co-incubated with each antibody treatment (antibody concentration, 25ug/mL; rifampin concentration 1ug/mL) prior to macrophage infection. Growth curves in the presence of each antibody treatment were generated by taking luminescence readings every 24 hours up to 96 hours. Y-axis (*Mtb* survival) is the area under the *Mtb* growth curve normalized by the no antibody condition of each donor. Each point is the triplicate average from 1 donor. Each donor is represented by a unique marker shape. Error bars, mean with standard deviation. Repeated measures one-way ANOVA with Sidak’s multiple comparisons test. P-values of relevant comparisons are shown. Source data

Because an antimicrobial role for polyclonal IgG and monoclonal IgG and IgA antibodies against *Mtb* has been proposed^19,28^, we next investigated whether IgM may also harbor antimicrobial capacity against *Mtb*. We engineered a high-affinity LAM-specific clone (A194)^29^ as IgG1 and IgM antibodies and compared the antimicrobial activity of each isotype in a human MDM model. Neither LAM-specific monoclonal antibody drove significant *Mtb* restriction in MDMs when added before (Extended Data Fig. 3) or following macrophage infection (Fig. 6a). While macrophages represent a primary cellular niche for *Mtb* in vivo during infection, we also probed the antimicrobial role of each antibody in a whole-blood model of infection, a system which queries the broader role of multiple immune cell types and components in microbial restriction. Fresh blood samples from healthy human donors (*n* = 4–6) were treated with the A194 LAM-specific IgG1 and IgM antibodies at the time of infection with *Mtb-276*, a luciferase reporter strain for *Mtb* in the H37Rv genetic background^30^. Luminescence readings were then taken every 24 h for 5 d to obtain the growth curves of *Mtb* in the presence of each antibody treatment. While the A194 LAM-specific IgG1 antibody was unable to drive significant *Mtb* restriction compared to its respective isotype control, the A194 LAM-specific IgM antibody drove significant restriction of *Mtb* growth, exhibiting a significantly lower area under the growth curve compared to both the IgM isotype control and the A194 IgG1 antibody (Fig. 6b). These data demonstrate that a high-affinity LAM-specific IgM antibody drove *Mtb* restriction in whole blood, suggesting that in addition to representing an early marker of vaccine-induced protection against TB, *Mtb*-specific IgM antibodies have the potential to functionally contribute to immunologic control of *Mtb*.

## Discussion

Here we probed the antibody response across multiple BCG vaccine regimens in rhesus macaques to determine whether specific antibody profiles are associated with *Mtb* control following vaccination. Elevated antigen-specific antibody titers were observed in both the plasma and the lungs of macaques vaccinated i.v. with BCG, with a marked expansion of functional and antimicrobial responses in the BAL. Correlation analyses revealed a particularly strong association of IgM responses in the plasma (weeks 8 and 24) and BAL (weeks 4 and 16) with reduced *Mtb* burden in BCG-vaccinated macaques. Finally, a LAM-specific IgM antibody resulted in enhanced restrictive activity of *Mtb* in vitro compared to the same antibody clone with an IgG1 heavy chain, pointing to a potential role for *Mtb*-specific IgM as a mechanistic correlate^31^ of vaccine-induced protection against *Mtb*.

IgG antibody titers represent the primary correlate of protection for the majority of approved vaccines^32^. Yet, vaccine-specific IgM titers were the only plasma antibody features to significantly negatively correlate with *Mtb* burden in immunized macaques. IgM did not represent an independent correlate of protection in this study because protection was so dominantly associated with the vaccine regimen. However, the potential value of IgM as a biomarker/correlate of immunity was corroborated by an enrichment in plasma IgM antibodies in macaques with enhanced control of *Mtb* following an orthogonal vaccine approach, driven by a.e. *Mtb-ΔsigH* immunization.

IgM has a critical role in the defense against encapsulated bacteria by driving phagocytosis, agglutination and complement activation^33,34^, and is a regulator of T cell responses^35^, suggesting that IgM may harbor both direct antimicrobial and indirect cellular regulatory roles in immunity against *Mtb*. We observed that a high-affinity LAM-specific IgM antibody restricted *Mtb* growth in whole-blood, but not in macrophages alone. The restrictive effect observed in whole-blood might reflect the contribution of additional immune factors in this assay, such as neutrophils, dendritic cells and/or complement. However, unlike the IgM antibodies elicited by vaccination or infection, the monoclonal IgM antibody used in these assays is an engineered form of a relatively high-affinity IgG antibody (A194-01)^29^. It is unclear whether the IgM antibodies generated by i.v. BCG or a.e. *Mtb-ΔsigH* immunization require a similarly high affinity to drive an antimicrobial response.

BCG immunization by the i.v. route was also uniquely associated with a significant increase in BAL IgG1, IgA and IgM titers to all mycobacterial antigens tested. It remains undefined whether the enhanced titers and function of the antibodies in the BAL at week 4 after vaccination represent a signature of protection, a signature of i.v. vaccination, or both. Follow-up studies with lower doses of BCG by i.v. injection are required to clarify the correlates of immunity against *Mtb*. Of note, repeated low-dose endobronchial BCG instillation showed increased protection against *Mtb* challenge, and significantly higher PPD-specific IgA and PPD-specific pan-isotype antibody titers in the BAL compared to standard i.d. BCG in macaques^36^, indicating that robust lung-localized antibody responses may be a common signature of protection. Both i.v. and endobronchially instilled BCG drive substantial BCG deposition in the lungs^11^, suggesting that the localization of vaccine antigen deep in lungs, rather than in the dermis or the upper respiratory tract could be critical for the induction of lung-specific immunity.

Unlike the T cell responses in the BAL, which did not contract over 24 weeks^11^, antibodies were less abundant in the BAL at week 16, and possibly even lower at the time of *Mtb* challenge (week 24, not measured). Nevertheless, select antibody features, including LAM-specific IgG1, IgA and IgM titers, as well as LAM-specific FcγR3A-binding antibodies, remained significantly higher in the airways of the i.v. BCG group at week 16 compared to the standard-dose i.d. BCG group. Given the small number of bacteria used in the challenge (10 colony-forming units (CFUs))^11^, and the limited number of bacteria believed to cause infection in humans^37^, even low titers of antibodies at the site of infection may serve as a first-line defense. Furthermore, work in the context of influenza infections has shown that vaccine-induced lung-resident memory B cells, particularly IgM^+^ memory B cells, may have a critical role in the rapid anamnestic response to infection resulting in the generation of antibody-secreting cells that repopulate the lung with antibodies able to control and clear infection^38,39^. Thus, it is possible that the antibodies detected in the airways at week 4 after vaccination may be a marker of lung-resident B cell immunity, which could respond at the time of *Mtb* challenge and contribute to protection.

At least three mechanisms of protection induced by i.v. BCG are possible: cell-mediated immunity^11^, humoral immunity and innate training of myeloid cells^40,41^. However, various immune responses may also work together to mediate protection. For example, antibodies augment cell-mediated immunity by engaging Fc receptors on antigen-presenting cells and driving a variety of downstream effects such as enhanced dendritic cell maturation, enhanced antigen presentation and co-stimulatory molecule upregulation, all of which may amplify T cell responses^42–44^. Further, while a cross-talk between antibody responses and BCG-induced innate training^40,41^ has not been thoroughly assessed, the hallmark of innate training is the increased responsiveness due to sustained changes in transcriptional programs through epigenetic reprogramming^40,41^. Thus, it remains plausible that i.v. BCG vaccination educates hematopoietic stem cells as described previously^40,41^, and renders macrophages uniquely poised to efficiently respond to and eliminate immune-complexed *Mtb* upon exposure. Future work should examine autologous antibody–macrophage interactions to dissect potential antibody–myeloid cell coordination during i.v. BCG vaccination.

Because of the potential safety issues associated with i.v. immunization, the development of alternative vaccination strategies able to mimic the protective immunity elicited by i.v. administration of BCG may be needed. Thus, this work demonstrates the value of comprehensive immune profiling across TB vaccine platforms, and motivates the continued study of antibodies, particularly IgM antibodies, as markers and as functional mediators of protection against TB.

## Methods

### Study design

Rhesus macaque (*Macaca mulatta*) plasma and BAL samples from the BCG-route vaccination cohort were collected during a study performed at the Vaccine Research Center at the National Institutes of Health^11^. All experimentation and sample collection from the original study complied with ethical regulations at the respective institutions^11^. In total, 48 BCG-immunized macaques were included in the study: 10 macaques received standard-dose i.d. BCG vaccination (target dose, 5 × 10^5^ CFUs), 8 macaques received high-dose i.d. BCG vaccination (target dose, 5 × 10^7^ CFUs), 10 macaques received i.v. BCG vaccination (target dose, 5 × 10^7^ CFUs), 10 macaques received a.e. BCG vaccination (target dose, 5 × 10^7^ CFUs) and 10 macaques received a combination of a.e. and standard-dose i.d. (a.e./i.d.) BCG vaccination (target dose, a.e. 5 × 10^7^ CFUs and i.d. 5 × 10^5^ CFUs)^11^. Twenty-four weeks following BCG vaccination, each macaque was challenged with 10 CFUs of *Mtb Erdman*, with a study endpoint of 12 weeks following *Mtb* challenge (Fig. 1a)^11^. In this study, *Mtb* burden values used throughout represent total thoracic CFUs measured at necropsy in the original study, and were measured as described previously^11^. Plasma samples were available and analyzed from the following time points: before vaccination, week 8 after BCG vaccination, time of challenge (week 24 after BCG vaccination) and week 28 (4 weeks after *Mtb* challenge; Fig. 1a). BAL samples were available and analyzed from the following time points: before vaccination, week 4 after BCG vaccination and week 12/16 after BCG vaccination (Fig. 1a). BAL samples from week 12 rather than week 16 were available and analyzed in four of eight macaques in the high-dose i.d. BCG vaccination group. BAL samples from all remaining macaques were available and analyzed at week 16. Post-*Mtb* challenge BAL samples were not collected, and thus were not available for antibody profiling. BAL fluid was received as a 10× concentrate, and further diluted for experiments.

Rhesus macaque plasma samples from the attenuated *Mtb* vaccination cohort were collected during a study performed at the Tulane National Primate Research Center^27^. All experimentation and sample collection from the original study were approved by the Institutional Animal Care and Use Committee and were performed in strict accordance with National Institutes of Health guidelines^27^. Plasma samples from nine rhesus macaques were analyzed in the present study. Four macaques received a.e. BCG vaccination (target dose, 1,000 CFUs), and five macaques received a.e. *Mtb-ΔsigH*—an attenuated *Mtb* strain in the CDC1551 genetic background—vaccination (target dose, 1,000 CFUs). Eight weeks after vaccination, each macaque was challenged with a target dose of 1,000 CFUs of *Mtb CDC1551* (ref. ^27^). Plasma samples from the following time points were analyzed: before vaccination, week 7 after vaccination and necropsy (week 15).

### Antigens

To profile humoral immune responses, a panel of BCG/*Mtb* antigens was used: PPD (Statens Serum Institute), HspX (provided by T. Ottenhoff), LAM (BEI Resources, NR-14848), PstS1 (BEI Resources, NR-14859) and Apa (BEI Resources, NR-14862). Zaire ebolavirus glycoprotein (R&D Systems) was used as a negative control for the attenuated *Mtb* analysis.

### Nonhuman primate reagents

Mouse anti-rhesus IgG1 (clone 7H11) and IgA (clone 9B9) secondary antibodies were obtained from the National Institutes of Health Nonhuman Primate Reagent Resource supported by grants AI126683 and OD010976. Mouse anti-monkey IgM (clone 2C11-1-5) was acquired from Life Diagnostics. Soluble rhesus macaque FcγR2A and FcγR3A were acquired from the Duke Human Vaccine Institute Protein Production Facility.

### Human research participants

Primary immune cells used in functional and antimicrobial assays were derived from the blood of healthy, HIV-negative individuals. These specimens were provided coded or anonymized. All donors provided written, informed consent, and the study was approved by the institutional review board at Massachusetts General Hospital.

### Antigen-specific antibody levels

Magnetic carboxylated fluorescent beads of distinct regions (Luminex) were first coupled to each protein antigen in a two-step carbodiimide reaction as described previously^12^. LAM was modified by 4-(4,6-dimethoxy[1,3,5]triazin-2-yl)-4-methyl-morpholinium (DMTMM) and coupled to Luminex magnetic carboxylated fluorescent beads using protocols described previously^45,46^.

Luminex using antigen-coupled beads to measure relative levels of antigen-specific antibodies was then performed as described previously^12^, with minor modifications. A master mix of antigen-coupled beads was made at a concentration of 16.67 beads per µl per region in 0.1% BSA-PBS, and 750 beads per region per well (45 µl) were added to a clear, flat-bottom 384-well plate (Greiner). Next, 5 µl of diluted sample was then added to the wells. Plasma from the BCG-route vaccination study was diluted and run at 1:10 and 1:100 dilutions. The 1:100 dilution was utilized for LAM IgG1, Apa IgG1, LAM IgA and all IgM antigens. The 1:10 dilution was used for the remaining conditions. BAL from the BCG-route vaccination study was diluted as follows: IgG1 1×, IgA 1× and IgM 0.1×. Plasma from the attenuated *Mtb* vaccination study was diluted as follows: IgG1, 1:150; IgA, 1:150; and IgM, 1:750. After adding the diluted samples, the plate was incubated with shaking at 700 r.p.m. overnight at 4 °C. Next, the plate was washed six times and 45 µl of mouse anti-rhesus IgG1, IgA or IgM antibody at 0.65 µg ml^−1^ was added, and incubated with shaking at 700 r.p.m. at room temperature (RT) for 1 h. The plate was then washed six times and 45 µl of phycoerythrin (PE)-conjugated goat anti-mouse IgG was added (Thermo Fisher, 31861) and incubated with shaking at 700 r.p.m. at RT for 1 h. The plate was then washed six times, and resuspended in Sheath Fluid (Luminex) in a final volume of 60 µl. PE MFI levels were then measured via the FlexMap 3D (Luminex). Data are represented as fold change over pre-vaccination levels. Samples were measured in duplicate.

### Antigen-specific Fcγ receptor binding

Rhesus macaque FcγRs were biotinylated as described previously^47^. In brief, each FcγR was biotinylated using a BirA biotin–protein ligase bulk reaction kit (Avidity) according to the manufacturer’s protocol, and excess biotin was removed using 3-kDa-cutoff centrifugal filter units (Amicon).

Luminex using biotinylated rhesus macaque FcγRs and antigen-coupled beads to measure relative binding levels of antigen-specific antibodies to FcγRs was then performed as described previously^47^, with minor modifications. A master mix of antigen-coupled beads was made at a concentration of 16.67 beads per µl per region in 0.1% BSA-PBS, and 750 beads per region per well (45 µl) were added to a clear, flat-bottom 384-well plate (Greiner). Next, 5 µl of sample (plasma: FcγR2A 1:10, FcγR3A 1:10; BAL: FcγR2A 1×, FcγR3A 1×) was added to the wells and incubated with shaking at 700 r.p.m. overnight at 4 °C. After overnight incubation, streptavidin-PE (ProZyme) was added to each biotinylated FcγR in a 4:1 molar ratio and incubated with rotation for 10 min at RT. Then, 500 µM biotin was then added at a 1:100 ratio relative to the total solution volume to quench the extra streptavidin-PE, and incubated with rotation for 10 min at RT. After washing the assay plate six times, 40 μl of each prepared detection FcγR (1 µg ml^−1^ in 0.1% BSA-PBS) was added to the immune-complexed microspheres and incubated with shaking at 700 r.p.m. for 1 h at RT. The plate was then washed six times, and resuspended in Sheath Fluid (Luminex) in a final volume of 60 µl. PE MFI levels were then measured via the FlexMap 3D (Luminex). Data are represented as fold change over pre-vaccination levels. Samples were measured in duplicate.

### Antibody-dependent cellular phagocytosis

**Extended Data Fig. 4:**
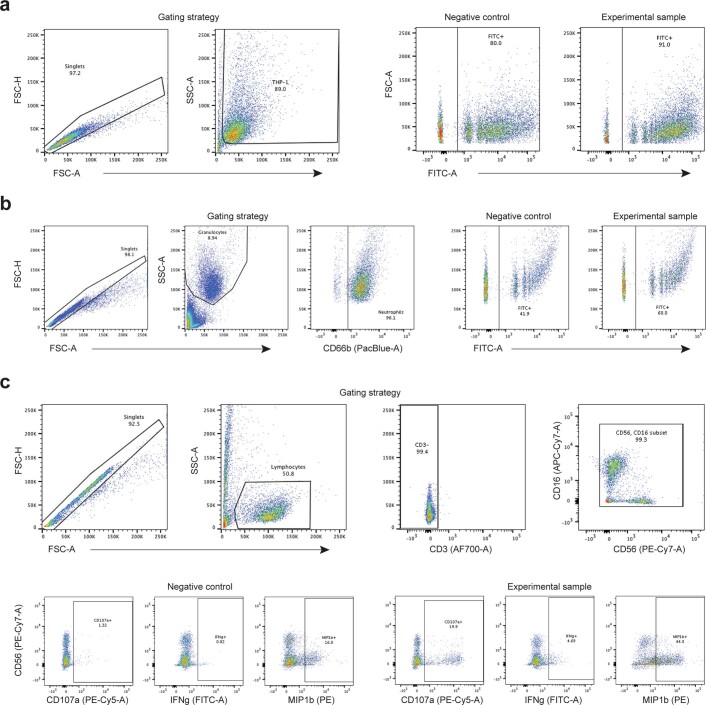
Gating strategies for flow-based antibody functional assays. **a**, Antibody-dependent cellular phagocytosis (ADCP). **b**, Antibody-dependent neutrophil phagocytosis. **c**, Antibody-dependent NK cell activation (ADNKA). Source data

PPD ADCP was measured as described previously^25^. LAM ADCP was measured as described previously^25^ with minor changes. For every 100 µg of LAM (dissolved in ddH_2_O), 10 µl of 1 M sodium acetate (NaOAc) and 2.2 µl of 50 mM sodium periodate (NaIO_4_) was added. This oxidation reaction proceeded for 45–60 min on ice in the dark. next, 12 µl of 0.8 M NaIO_4_ was then added to block oxidation, and the solution was incubated for 5 min at RT in the dark. Next, the oxidized LAM was transferred to a new tube, and 10 µl of 1 M NaOAc and 22 µl of 50 mM hydrazide biotin (Sigma) were added. This biotinylation reaction proceeded for 2 h at RT. Excess biotin was then removed using Amicon Ultra 0.5-ml columns (3K, Millipore Sigma) according to the manufacturer’s instructions. Biotinylated LAM was then added to FITC-conjugated neutravidin beads (Invitrogen, 1.0 µm) at a ratio of 1 µg antigen:4 µl beads, and incubated overnight at 4 °C. Excess antigen was washed away. Antigen-coated beads were incubated with 10 µl of sample (plasma 1:10, BAL 1:1) for 2 h at 37 °C. THP-1 cells (5 × 10^4^ per well) were added and incubated at 37 °C for 16 h. Bead uptake was measured in fixed cells using flow cytometry on a BD LSR II (BD Biosciences). Data were collected in FACSDiva (version 9.0) and analyzed using FlowJo 10.3 (Extended Data Fig. 4). Phagocytic scores were calculated as: ((% FITC-positive cells) × (geometric mean fluorescence intensity of the FITC-positive cells)) divided by 10,000. Data are represented as fold change over pre-vaccination levels. Samples were run in duplicate.

### Antibody-dependent neutrophil phagocytosis

ADNP was performed as described previously^48^, with minor changes. LAM was biotinylated and coupled to fluorescent neutravidin beads (1.0 µm, Invitrogen), incubated with serum and washed as described above for ADCP. During the 2-h bead and serum incubation, fresh peripheral blood collected from healthy donors in acid citrate dextrose anticoagulant tubes was added at a 1:9 ratio to ACK lysis buffer (150 mM NH_4_Cl, 8,610 mM KHCO_3_, 0.1 mM Na_2_-EDTA, pH 7.4) for 5 min at RT. After red blood cell lysis, the blood was centrifuged for 5 min at 1,500 r.p.m. After centrifugation, supernatant was removed, and leukocytes were washed with 50 ml of 4 °C PBS, spun for 5 min at 1,500 r.p.m. and resuspended in R10 medium (RPMI (Sigma), 10% fetal bovine serum (Sigma), 10 mM HEPES (Corning), 2 mM l-glutamine (Corning)) at a final concentration of 2.5 × 10^5^ cells per ml. Leukocytes (5 × 10^4^ cells per well) were then added to the immune-complexed beads and incubated for 1 h at 37 °C 5% CO_2_. Following this incubation, the plates were spun for 5 min at 500*g*. After removing the supernatant, antihuman CD66b-Pacific Blue (BioLegend) was diluted at a ratio of 1:75 and added to the leukocytes before incubation for 20 min at RT. Following this incubation, the cells were washed with PBS and fixed. Bead uptake was measured in fixed cells using flow cytometry on a BD LSR II (BD Biosciences). Data were collected in FACSDiva (version 9.0) and analyzed using FlowJo 10.3 (Extended Data Fig. 4). Phagocytic scores were calculated in the CD66b-positive cell population. Data are represented as fold change over pre-vaccination level. Samples were run in duplicate.

### Antibody-dependent NK cell activation

Antibody-dependent NK cell activation was performed as described previously^25^, with minor changes. ELISA plates (Thermo Fisher, NUNC MaxiSorp flat bottom) were coated with 150 ng per well of LAM and incubated overnight at 4 °C. The plates were then washed with PBS and blocked with 5% BSA-PBS for 2 h. Next, the plates were washed with PBS, and 50 µl of sample (plasma 1:10, BAL 1×) was added and incubated for 2 h at 37 °C. One day before adding the diluted sample, NK cells were isolated from healthy donors using the RosetteSep human NK cell enrichment cocktail (Stemcell) and Sepmate conical tubes (Stemcell) according to the manufacturer’s instructions. Following isolation, NK cells were incubated overnight at 1.5 × 10^6^ cells per ml in R10 media with 1 ng ml^−1^ human recombinant IL-15 (Stemcell). After the 2-h serum incubation, the assay plates were washed, and 50,000 primary human NK cells, together with 2.5 µl PE-Cy5 antihuman CD107a (BD), 0.4 µl brefeldin A (5 mg ml^−1^, Sigma) and 10 µl GolgiStop (BD) were added to each well of the assay plates. The plates were then incubated for 5 h at 37 °C. Following the incubation, the samples from each well were stained with 1 µl each of: PE-Cy7 antihuman CD56 (1:10 dilution), APC-Cy7 antihuman CD16 (1:10 dilution) and Alexa Fluor 700 antihuman CD3 (1:10 dilution; all from BD). After a 20-min incubation at RT to allow extracellular staining, the plate was washed with PBS, and the cells were fixed using Perm A and Perm B (Invitrogen). The Perm B solution additionally contained PE antihuman MIP-1β (1:50 dilution) and FITC antihuman IFN-γ (1:20 dilution; both from BD) to allow intracellular cytokine staining. After a final wash in PBS, the cells were resuspended in PBS and the fluorescence of each marker was measured on a BD LSR II flow cytometer. Data were collected in FACSDiva (version 9.0) and analyzed using FlowJo 10.3 (Extended Data Fig. 4). Data are represented as fold change over pre-vaccination levels. The assay was performed in biological duplicate using NK cells from two different donors.

### Macrophage restriction assay (*Mtb*-live/dead)

In vitro macrophage *Mtb* survival was measured as described previously^25^, with minor changes. CD14-positive cells were isolated from HIV-negative donors using the EasySep CD14 Selection Kit II according to the instructions of the manufacturer (Stemcell). CD14-positive cells were matured for 7 d in R10 media without phenol in low-adherent flasks (Corning). MDMs were plated at 50,000 cells per well in glass-bottom, 96-well plates (Greiner) 24 h before infection. A reporter *Mtb* strain in the H37Rv genetic background with constitutive mCherry expression and inducible GFP expression (*Mtb*-live/dead)^26^, was cultured in log phase and filtered through a 5-μm filter (Milliplex) before MDM infection at a multiplicity of infection of 1 for 14 h at 37 °C. Extracellular bacteria were washed off, and 200 µl of pooled sample from each of the vaccination groups diluted in R10 without phenol (plasma 1:100, BAL 1×) was added. Three days following infection, anhydrotetracycline (Sigma) (200 ng ml^−1^) was added for 16 h to induce GFP expression. Ninety-six hours following infection, cells were fixed and stained with DAPI. Data were analyzed using the Columbus Image Data Storage and Analysis System and Microsoft Excel (version 16.43). Bacterial survival was calculated as the ratio of live to total bacteria (the number of GFP-positive pixels (live) divided by the number of mCherry-positive pixels (total burden)) within macrophages in each well. Bacterial survival for each condition was normalized by bacterial survival in the no-antibody condition. The assay was performed in technical triplicate using MDMs from four different donors.

### Macrophage restriction assays (*Mtb-276*)

CD14-positive cells were isolated from HIV-negative donors using the EasySep CD14 Selection Kit II according to the instructions of the manufacturer (Stemcell). CD14-positive cells were matured for 7 d in R10 media without phenol in low-adherent flasks (Corning). MDMs were plated 50,000 cells per well in sterile, white, flat-bottom 96-well plate plates (Greiner) 24 h before infection. *Mtb-276*, a luciferase reporter strain for *Mtb* in the H37Rv genetic background^30^, was cultured in log phase and filtered through a 5-μm filter (Milliplex). In the pre-infection model (Extended Data Fig. 3), *Mtb-276* was co-incubated with each antibody treatment in R10 (final antibody concentration of 25 µg ml^−1^) for 1 h at 37 °C before infection at a multiplicity of infection of 1. In the post-infection model (Fig. 6), MDMs were infected at a multiplicity of infection of 1, for 14 h at 37 °C. Next, extracellular bacteria were washed off, and each antibody treatment was diluted to 50 µg ml^−1^ in R10 and added to the infected MDMs. Control treatments: 1 µg ml^−1^ rifampin (Sigma), human IgG1 isotype control (BE0297, BioXcell), and human IgM isotype control (31146, Invitrogen). In the pre-infection and post-infection models, luminescence readings were taken every 24 h, up to 96 h following infection to obtain *Mtb* growth curves in the presence of each antibody treatment. Area under the curve values were then computed for each antibody treatment in GraphPad Prism (version 8.4.0).

### Whole-blood restriction assay

Whole-blood from HIV-negative human donors was collected fresh on the day of the experiment in acid citrate dextrose tubes. *Mtb-276*, a luciferase reporter strain for *Mtb* in the H37Rv genetic background^30^, previously cultured in 7H9 media at 37 °C in log phase was washed once and resuspended in R10 media without phenol. Whole blood was then infected with *Mtb-276* such that the final concentration was 1 × 10^6^ bacteria per ml of blood. Immediately after adding *Mtb-276* to blood, 150 µl of blood and 150 µl of antibody samples pre-diluted to 50 µg ml^−1^ in R10 media were added together into a sterile, white, flat-bottom 96-well plate in triplicate (Greiner). The final concentration of experimental antibody treatments was 25 µg ml^−1^, and the final concentration of control treatments was: rifampin (Sigma) 0.25 µg ml^−1^, human IgG1 isotype control (BE0297, BioXcell) 25 µg ml^−1^ and human IgM isotype control (31146, Invitrogen) 25 µg ml^−1^. Samples in each well were mixed, and then the first luminescence reading was taken on a plate reader (Tecan Spark 10M). The plate was then incubated at 37 °C. Every 24 h post-infection for 120 h, the samples in each well were mixed, and luminescence readings were taken on a plate reader to obtain *Mtb* growth curves in the presence of different antibody treatments. *Mtb* restriction in whole blood was calculated as the area under the curve for each condition. Area under the curve values were computed for each antibody treatment in GraphPad Prism (version 8.4.0).

### LAM-specific monoclonal antibody expression

A194 LAM-specific antibodies were generated as described previously^29^. In brief, A194 IgG1 was generated by transfecting the A194-IGG1VH and IGVK plasmids into Expi293 cells. A194-IgM was generated by transfecting the A194-IGM1VH, IGVK and joining (J) chain plasmids into Expi293 cells to generate multimeric IgM. Each antibody was purified by affinity chromatography. Protein A beads and protein L beads were used for the purification of IgG1 and IgM respectively. The antibodies were eluted using a low-pH buffer, and characterized by SDS–PAGE for purity and size.

### Partial least-squares discriminant analysis

A multivariate model to distinguish protected and susceptible macaques was generated using a combination of LASSO-based feature selection, and partial least-squares discriminant analysis (PLS-DA). Protected macaques were defined as those with an *Mtb* burden less than 1,000 CFUs per ml at time of necropsy. *Mtb* burden values used represent total thoracic CFUs measured at necropsy in the original study, and were measured as described previously^11^.

For feature selection, the data were *z*-scored and 100 bootstrap datasets were generated. A LASSO model in which the optimal penalty term lambda was chosen via fivefold cross-validation and was then fit on each bootstrap dataset, and coefficients from each iteration of LASSO regularization were stored. Using these coefficients, variable inclusion probabilities—defined as the proportion of bootstrap replications in which a coefficient estimate is not zero—were computed for each antibody feature. LASSO regularization was implemented using the glmnet package (version 3.0-2) in R (version 3.6.2).

PLS-DA models across a grid of variable inclusion probability cutoffs were fit in a fivefold cross-validation framework repeated 100 times. Model accuracy—defined as ((1 − balanced error rate) × 100)—was computed for each model. The optimal model, which contained three antibody features, was found at a variable inclusion probability of 0.45. A graph of the first and second LV from the optimal PLS-DA model was included, as was a VIP plot, indicating the relative contribution of individual features to separation along the first LV. The significance of the model was assessed using a permutation test. Specifically, the group labels of the macaques were randomly permuted. PLS-DA models were then fit and evaluated for model accuracy in a fivefold cross-validation framework repeated 100 times. The accuracy of the real model was compared with that of the permuted model using a two-tailed Mann–Whitney *U* test. PLS-DA models were implemented using the mixOmics package^49^ (version 6.10.9) in R (version 3.6.2).

### Statistics

For the antibody titer, FcγR binding, and functional measurements, from the BCG vaccination cohort, Kruskal–Wallis with Dunn’s multiple-comparison tests were performed on the fold change values at each time point, comparing each vaccination group to the standard-dose i.d. BCG group. For the macrophage *Mtb* restriction assay on the BCG vaccination cohort, a repeated-measures one-way ANOVA with Dunnett’s multiple-comparisons test was performed for each vaccination group, comparing pre-vaccination restrictive activity with that of each post-vaccination time point. For antibody titers in the attenuated *Mtb* vaccination cohort (*Mtb-ΔsigH*), two-tailed Mann–Whitney *U* tests were performed on the fold change values at each time point, comparing *Mtb-ΔsigH* to the BCG group. For the LAM-specific monoclonal antibody *Mtb* restriction assays in macrophages, a repeated-measures one-way ANOVA with Sidak’s multiple-comparisons test was performed. For the whole-blood assay, an ordinary one-way ANOVA with Sidak’s multiple-comparisons test was performed. These statistics were performed in GraphPad Prism (version 8.4.0). Spearman correlations between *Mtb* burden and individual antibody features were computed in R (version 3.6.2). Adjusted *P* values (*q* values) were calculated using the Benjamini–Hochberg procedure^50^.

### Reporting Summary

Further information on research design is available in the Nature Research Reporting Summary linked to this article.

## Online content

Any methods, additional references, Nature Research reporting summaries, source data, extended data, supplementary information, acknowledgements, peer review information; details of author contributions and competing interests; and statements of data and code availability are available at 10.1038/s41590-021-01066-1.

## Supplementary information

Reporting Summary

Supplementary Data 1 Systems serology data.

## Data Availability

Source data are provided with this paper. All other data and metadata associated with this study are available in the main text, Supplementary Information and/or at https://fairdomhub.org/studies/876.

## Source data

Source Data Fig. 1 Statistical source data.
Source Data Fig. 2 Statistical source data.
Source Data Fig. 3 Statistical source data.
Source Data Fig. 4 Statistical source data.
Source Data Fig. 5 Statistical source data.
Source Data Fig. 6 Statistical source data.
Source Data Extended Data Fig. 1 Statistical source data.
Source Data Extended Data Fig. 2 Statistical source data.
Source Data Extended Data Fig. 3 Statistical source data.
Source Data Extended Data Fig. 4 Statistical source data.

## Code availability

Scripts to reproduce the computational analyses presented in the paper are available at https://github.com/eirvine94/IVBCG_antibody_manuscript/.
