# Supplementary material for: Robust IgM responses following intravenous vaccination with Bacille Calmette–Guérin associate with prevention of Mycobacterium tuberculosis infection in macaques
Source: Nat Immunol. 2021 Nov 22;22(12):1515–23. doi: 10.1038/s41590-021-01066-1 (PMC8642241; doi:10.1038/s41590-021-01066-1)
Supplement: Supplementary file 1 — Reporting Summary [file 41590_2021_1066_MOESM1_ESM.pdf]

nature research

Corresponding author(s): Galit Alter and Sarah Fortune

Last updated by author(s): Sep 24, 2021

Reporting Summary

Nature Research wishes to improve the reproducibility of the work that we publish. This form provides structure for consistency and transparency in reporting. For further information on Nature Research policies, see our Editorial Policies and the Editorial Policy Checklist.

Statistics

For all statistical analyses, confirm that the following items are present in the figure legend, table legend, main text, or Methods section.

n/a	Confirmed	
☐	☒	The exact sample size (*n*) for each experimental group/condition, given as a discrete number and unit of measurement
☐	☒	A statement on whether measurements were taken from distinct samples or whether the same sample was measured repeatedly
☐	☒	The statistical test(s) used AND whether they are one- or two-sided<br>*Only common tests should be described solely by name; describe more complex techniques in the Methods section.*
☐	☒	A description of all covariates tested
☐	☒	A description of any assumptions or corrections, such as tests of normality and adjustment for multiple comparisons
☐	☒	A full description of the statistical parameters including central tendency (e.g. means) or other basic estimates (e.g. regression coefficient) AND variation (e.g. standard deviation) or associated estimates of uncertainty (e.g. confidence intervals)
☐	☒	For null hypothesis testing, the test statistic (e.g. *F*, *t*, *r*) with confidence intervals, effect sizes, degrees of freedom and *P* value noted<br>*Give P values as exact values whenever suitable.*
☒	☐	For Bayesian analysis, information on the choice of priors and Markov chain Monte Carlo settings
☒	☐	For hierarchical and complex designs, identification of the appropriate level for tests and full reporting of outcomes
☐	☒	Estimates of effect sizes (e.g. Cohen's *d*, Pearson's *r*), indicating how they were calculated

Our web collection on statistics for biologists contains articles on many of the points above.

Software and code

Policy information about availability of computer code

Data collection	FACSDiva version 9.0
Data analysis	GraphPad Prism (version 8.4.0); R (version 3.6.2); Microsoft Excel (version 16.43); FlowJo (version 10.3); Columbus Image Data Storage and Analysis System<br><br>R packages used: glmnet (version 3.0-2); mixOmics (version 6.10.9)<br><br>Custom code to reproduce computational analyses available at: https://github.com/eirvine94/IVBCG_antibody_manuscript

For manuscripts utilizing custom algorithms or software that are central to the research but not yet described in published literature, software must be made available to editors and reviewers. We strongly encourage code deposition in a community repository (e.g. GitHub). See the Nature Research guidelines for submitting code & software for further information.

Data

Policy information about availability of data

All manuscripts must include a data availability statement. This statement should provide the following information, where applicable:

- Accession codes, unique identifiers, or web links for publicly available datasets
- A list of figures that have associated raw data
- A description of any restrictions on data availability

All data associated with this study are available in the main text, source data, or supplemental data.

Field-specific reporting

Please select the one below that is the best fit for your research. If you are not sure, read the appropriate sections before making your selection.

☒ Life sciences ☐ Behavioural & social sciences ☐ Ecological, evolutionary & environmental sciences

For a reference copy of the document with all sections, see nature.com/documents/nr-reporting-summary-flat.pdf

Life sciences study design

All studies must disclose on these points even when the disclosure is negative.

Sample size	Sample size was dictated by the number of animals included in the original vaccination study (PMC7015856).
Data exclusions	No data were excluded from the analyses.
Replication	All the reported experiments were reproducible. Systems Serology measurements were captured in duplicate; technical replicates were minimally different. Mtb infection assays were performed in triplicate using cells from at least 4 healthy human donors.
Randomization	Rhesus macaques from the original BCG vaccination study were randomized into experimental groups based on gender, weight and pre-vaccination T cell responses to PPD (Darrah...Seder Nature 2020 | PMC7015856). Randomization did not apply to monoclonal antibody experiments.
Blinding	Investigators were blinded to vaccination group during Systems Serology profiling. Investigators were not blinded during monoclonal antibody experiments.

Reporting for specific materials, systems and methods

We require information from authors about some types of materials, experimental systems and methods used in many studies. Here, indicate whether each material, system or method listed is relevant to your study. If you are not sure if a list item applies to your research, read the appropriate section before selecting a response.

Materials & experimental systems

n/a	Involved in the study	
☐	☒	Antibodies
☐	☒	Eukaryotic cell lines
☒	☐	Palaeontology and archaeology
☒	☐	Animals and other organisms
☐	☒	Human research participants
☒	☐	Clinical data
☒	☐	Dual use research of concern

Methods

n/a	Involved in the study	
☒	☐	ChIP-seq
☐	☒	Flow cytometry
☒	☐	MRI-based neuroimaging

Antibodies

Antibodies used

National Institutes of Health Nonhuman Primate Reagent Resource:
anti-rhesus IgG1 | clone 7H11 | lot 013119EP| cat AB_2819310
anti-rhesus IgA | clone 9B9 | lot 102510X | cat AB_2819305

Life Diagnostics:
anti-monkey IgM |clone 2C11-1-5 | lot C-l1515A | cat 2C11-1-5

ThermoFisher:
PE anti-mouse IgG | RRID AB_429715 | lot VC2960991 | cat 31861
Human IgM isotype control | clone N/A | lot VE3001873 | cat 31146

BioLegend:
anti-human CD66b-Pacific Blue | clone G10F5 | lot B256448| cat 305112

BD Biosciences:
PE-Cy7 anti-human CD56 | clone B159 | lot 0274120 | cat 557747
APC-Cy7 anti-human CD16 | clone 3G8 | lot 9289979| cat 557758
Alexa Fluor 700 anti-human CD3 | clone UCHT1 | lot 7145618| cat 557943
PE anti-human MIP-1β | clone D21-1351 | lot 0065243 | cat 550078
FITC anti-human IFNγ | clone 25723.11 | lot 0342682| cat 340449
PE-Cy5 anti-human CD107a | clone H4A3 (RUO) | lot 0149826 | cat 555802

	BioXcell:<br>Human IgG1 isotype control | clone N/A | lot 659518A1 | cat BE0297
Validation	All antibodies were used according to manufacturer's instructions and previously published methods. They were validated and titrated for specificity prior to use.<br><br>Antibody-dependent neutrophil phagocytosis, validation of CD66b antibody described in: PMID 27667685, PMID 29605231, PMID 30096313, PMID 30092199 PMID 30029854, and PMID 30629918.<br><br>Antibody dependent NK cell activation, validation of antibodies described in: PMID 23468501, 2) PMID 24648341, PMID 26745376, PMID 27667685, PMID 30096313, PMID 30092199, and PMID 30029854.<br><br>anti-rhesus antibodies, validation described in: PMC7906955 and PMC8008062.

Eukaryotic cell lines

Policy information about cell lines

Cell line source(s)	THP1 from ATCC Cat#: TIB202; Expi293 from ThermoFisher Cat# A14527
Authentication	Cell lines commercially purchased. THP1 authentication was performed via STR Profiling service ATCC 135-XV.
Mycoplasma contamination	Negative for mycoplasma. THP1 cell line was tested and negative for mycoplasma via ATCC Univ Mycoplasma kit 30-1012K.
Commonly misidentified lines<br>(See ICLAC register)	No commonly misidentified cell lines were used.

Human research participants

Policy information about studies involving human research participants

Population characteristics	The samples used in this study are primary immune cells derived from the blood of healthy, HIV negative individuals. These specimens were provided coded or anonymized.
Recruitment	No demographic characteristics were used for selection. Selection was based on no clinical signs of illness and diagnostic testing negative for active HIV, HCV, and HBV infections.
Ethics oversight	All donors provided written, informed consent, and the study was approved by the institutional review board at Massachusetts General Hospital.

Note that full information on the approval of the study protocol must also be provided in the manuscript.

Flow Cytometry

Plots

Confirm that:

☒ The axis labels state the marker and fluorochrome used (e.g. CD4-FITC).

☒ The axis scales are clearly visible. Include numbers along axes only for bottom left plot of group (a 'group' is an analysis of identical markers).

☒ All plots are contour plots with outliers or pseudocolor plots.

☒ A numerical value for number of cells or percentage (with statistics) is provided.

Methodology

Sample preparation	Fresh peripheral blood was collected from healthy donors in acid citrate dextrose (ACD) anti-coagulant tubes.
Instrument	BD LSRII and FlexMap 3D.
Software	FACSDiva (version 9.0) and FlowJo (version 10.3)
Cell population abundance	Cell sorting not performed.
Gating strategy	See Extended Data Fig. 4. Gating strategy with FSC/SSC gates are included. Boundaries of negative and positive populations were determined based on unstained and fluorescence minus one control samples.

☒ Tick this box to confirm that a figure exemplifying the gating strategy is provided in the Supplementary Information.
